# Physician’s Pocket Account Book

**Published:** 1905-12

**Authors:** 


### Physician's Pocket Account Book.

The practical value of Taylor's pocket account book is evident to any one examining a copy. It is neat, compact, and easily kept. All entries are made but once and require no posting. There is nothing irregular or obscure about it, and it would readily be admissible in court as evidence. Published by J. J. Taylor, editor *Medical Council,* 4105 Walnut St., Philadelphia; bound in leather, $1.00.

---

A successful medical practitioner of many years standing makes the following statement: "There are a large majority of combinations which extemporaneous pharmacy can not prepare properly, and I know that through the dishonesty, ignorance or indifference of many retail druggists we are not able to get on prescriptions the very best drugs, hence it is to the manufacturing pharmacists, whose best interest lies in the purity and uniformity of his product, that we must look for our most reliable remedies. I endorse worthy proprietaries, but I most heartily condemn the great tendency of the half-baked, so-called manufacturing "chemist," to foist upon the profession and public cheap imitations of standard preparations."

